# Monitoring and Evaluation of Dementia-Friendly Neighborhoods Using a Walkshed Approach: Protocol for a Scoping Review

**DOI:** 10.2196/50548

**Published:** 2024-01-03

**Authors:** Mark Groulx, Shannon Freeman, Keone Gourlay, Dawn Hemingway, Emma Rossnagel, Habib Chaudhury, Mohammadjavad Nouri

**Affiliations:** 1 University of Northern British Columbia Prince George, BC Canada; 2 School of Nursing University of Northern British Columbia Prince George, BC Canada; 3 School of Planning and Sustainability University of Northern British Columbia Prince George, BC Canada; 4 School of Social Work University of Northern British Columbia Prince George, BC Canada; 5 Department of Gerontology Simon Fraser University Vancouver, BC Canada

**Keywords:** dementia-friendly, neighborhood, persons living with dementia, walkability, walkshed

## Abstract

**Background:**

The number of people in society living with dementia is growing. In Canada, most people who live with dementia live at home, often in a neighborhood setting. Neighborhood environments can be a source of independence, social engagement, and well-being. They can also contain barriers that limit physical activity, social engagement, and well-being. A dementia-friendly neighborhood includes assets that support persons living with dementia and their caregivers in multiple life domains, including those that support walking within the neighborhood environment.

**Objective:**

The objectives for this scoping review are twofold. First, focusing on walkshed analysis, we aim to extend scholarly understandings of methodological practices used in the monitoring and evaluation of dementia-friendly neighborhoods. Second, we aim to provide clear and practical guidance for those working in planning, design, and public health fields to assess the neighborhood context in support of evidence-based action to improve the lives of persons living with dementia.

**Methods:**

The study design follows Arksey and O’Malley’s scoping review framework and PRISMA-P (Preferred Reporting Items for Systematic Review and Meta-Analysis Protocols) guidelines. We will conduct a search of peer-reviewed studies in 6 electronic databases to identify the use of Geographic Information System analysis to measure the walkshed of persons living with dementia in a community setting. As age is a primary risk factor associated with dementia, we will also include studies that focus more broadly on community-dwelling older adults aged 65 years and older. Data will be extracted, analyzed, and represented according to 3 domains. This includes study details, walkshed analysis methods, and criteria and indicators used to measure dementia-friendly neighborhoods.

**Results:**

The results of the study and the submission of a manuscript for peer review are expected in June 2024. The results of the review are expected to contribute to an understanding of methods for monitoring and evaluating dementia-friendly neighborhoods. Expected findings will include a detailed breakdown of current parameters and routines used to conduct walkshed analysis. Findings will also convey criteria that can be operationalized in a Geographic Information System as indicators to assess barriers and facilitators to walking in a neighborhood setting.

**Conclusions:**

As far as we are aware, the proposed scoping review will be the first to provide comprehensive methodological or technical guidance for conducting walkshed analysis specific to persons living with dementia. Both the scalability and objective nature of walkshed analysis are likely to be of direct interest to public health practitioners, planners, and allied professionals. Clearly documenting methods used in walkshed analysis can spur increased collaboration across these disciplines to enable an evidence-informed approach to improving neighborhood environments for persons living with dementia.

**International Registered Report Identifier (IRRID):**

PRR1-10.2196/50548

## Introduction

### Overview

Walkshed analysis identifies the extent of the community environment surrounding a central location that is accessible at a scale where walking is a competitive mode of mobility [1]. Once a walkshed is delineated in a Geographic Information System (GIS), criteria and indicators can identify barriers and enablers to walking [2]. Walkshed analysis is relevant to planning and public-health partnerships that seek to support persons living with dementia. More than 55 million people are currently living with dementia across the world. The global prevalence of dementia is projected to continue to rise by approximately 10 million new cases per year [3]. In Canada, most people experiencing dementia live at home. As of 2016, around 69% of those aged 80 years or younger were living outside of the long-term care system [4]. As an umbrella term, dementia captures the experience of progressive cognitive decline. It can impact an individual’s mood, behavior, and actions, including the performance of key activities of daily living [5]. There are many types of dementia, including Alzheimer dementia, vascular dementia, frontotemporal dementia, lewy body dementia, mixed dementia, and young-onset dementia. Alzheimer disease is the most common cause, contributing to 60% to 70% of cases of dementia [6].

Literature on dementia-friendly communities (and neighborhoods) takes a relational view [7,8]. This view acknowledges that well-being is conditioned by interrelated aspects of a person’s social, built, and ecological surroundings [8-10]. Accordingly, scholars identify dementia-friendly environments as the arrangement of supportive assets into a community fabric that promotes meaningful societal engagement for persons living with dementia and their caregivers [11]. This includes the complex social relations that persons living with dementia experience in a community setting, making the physical neighborhood part of a relational and moral context [12,13].

Scholarship on dementia-friendly communities and neighborhoods stems from calls to better support persons who are living with dementia outside of an institutional setting [11]. These calls reflect the fact that scholars have long viewed neighborhoods as a central relational context shaping individual behavior and life quality [14]. As early as the turn of the 20th century, ideas about neighborhood planning in North America drew on sociological concepts such as Charles H Cooley’s primary group. The primary group and similar concepts asserted that the neighborhood was the main setting for the social relations that informed one’s perspectives and ideals [15].

A long fascination with neighborhood environments helps explain the growing effort to understand how the neighborhood can enable or hinder self-determination for persons living with dementia. This includes aspects of identity development and one’s ability to shape life balance [10,12,16]. Remaining close to the home, or aging in place, is also “closely intertwined with (a person’s) sense of self and identity” [17]. By contrast, moving away from familiar areas can have negative effects on persons living with dementia [18]. To remain active and engaged within their environments while aging in place, persons living with dementia need special considerations and support in their neighborhoods [17].

The influence of the built environment on a sense of community and one’s place therein remains up for debate in an increasingly mobile and digital society [19,20]. At the same time, there is a convincing body of evidence demonstrating that planning and design can impact behavior. The extent to which a neighborhood setting encourages or discourages important social and health behaviors such as walking is a particular focus for planning-health partnerships [21-23]. There is also a growing body of evidence illustrating that walking outdoors boosts quality of life for those living with dementia, contributing to improved mood, quality of sleep, and sense of freedom [17,24,25].

Urban planning scholar Lawrence Frank significantly advanced the conception and measurement of walkability. He describes walkability as the extent to which an environment’s social and physical characteristics promote walking as a competitive and desirable form of mobility [26,27]. Recent work has extended the idea of walkability to a more encompassing notion of “active living environments.” Active living environments are defined as “the emergent natural, built, and social properties of neighborhoods that promote physical activity and health and allow for equitable access to health-enhancing resources” [28].

Scholars have used a wide variety of methods to study walkability and its relation to walking behavior. These include phenomenological interviews [29], cross-sectional community surveys [30], observational techniques [31], surveys [32,33], photovoice [34,35], and in-situ walking interviews [12]. Scholars have also deployed criteria and indicators that enable monitoring and evaluation of the social, built, and ecological environments that make up a city [36]. In some cases, criteria and indicators are operationalized using a geospatial approach that assesses barriers and facilitators to walking in a small area (eg, 1 km) surrounding a central location such as a residence. This approach is often referred to as walkshed analysis.

In North America, walkability is now well researched within urban settings in the context of the “general population.” By comparison, factors that shape walkability for members of equity-deserving groups, particularly persons living with dementia, are comparatively understudied. There is a need to better document (1) what walkability criteria and indicators are relevant to the lived experience of persons living with dementia, (2) how methods are operationalized to examine barriers and facilitators using a walkshed approach, and (3) where barriers and facilitators of walkability for persons living with dementia may align or conflict with those of other populations. Given these needs, the objectives for this scoping review are twofold:

1. Focusing on walkshed analysis, extend scholarly understandings of methodological practices used in the monitoring and evaluation of dementia-friendly neighborhoods.
2. Provide clear and practical guidance for those working in planning, design, and public health fields to assess the neighborhood context in support of evidence-based action to improve the lives of persons living with dementia.

To achieve the preceding objectives, this scoping review will address the following research question: What dimensions, criteria, and indicators can be recognized within the academic literature for measuring neighborhood walkability for persons living with dementia based on a walkshed methodology?

### Existing Reviews

This protocol was informed by an initial review of existing peer-reviewed literature. The purpose of this review was to identify possible knowledge syntheses on the use of walkshed methodology to document barriers and facilitators faced by persons living with dementia. Table 1 summarizes key aspects of 6 related knowledge syntheses. All but 1 of the identified studies were published within the past 5 years [36]. A total of 2 of the studies directly focused on persons living with dementia. Other studies focused on dementia risk factors among older adults (see Table 1).

**Table 1:** Summary of comparable existing knowledge syntheses as they relate to the proposed scoping review.

Reference	Title	Objective	Population focus	Addresses aspects of walkshed methods	Addresses objective criteria
Akinci et al [21], 2022	How different are objective operationalizations of walkability for older adults compared to the general population? a systematic review	Summarize and compare methods used to operationalize objective walkability for older adults and the general population	Older adults or general population	Yes	Yes
Cerin et al [36], 2017	The neighbourhood physical environment and active travel in older adults: a systematic review and meta-analysis	Identify correlates of neighborhood physical features and active travel in older adults and quantify the strength of associations	Older adults	No	Yes
Sturge et al [37], 2021	Features of the social and built environment that contribute to the well-being of persons with dementia who live at home: a scoping review	Summarize evidence from qualitative studies about how social and built environment features influence well-being for persons living with dementia	Persons living with dementia	No	Yes
Gan et al [25], 2022	Dementia-friendly neighbourhood and the built environment: a scoping review	Synthesize knowledge and support policy direction related to dementia-friendly neighborhood environments and attendant psychosocial outcomes	Persons living with dementia	No	Yes
Peters et al [2], 2020	Measuring the association of objective and perceived neighborhood environment with physical activity in older adults: challenges and implications from a systematic review	Assess the correlates of neighborhood characteristics and physical activity in older adults to provide a body of evidence to support neighborhood environmental interventions	Older adults	Yes	Yes
Chen et al [38], 2022	Neighbourhood-built environment associated with cognition and dementia risk among older adults: a systematic literature review	Assess the state of current knowledge on the links between neighborhood environments and cognitive health in older adults	Older adults at risk of dementia	No	Yes

Gan and colleagues [25] reviewed 29 studies and documented methodologies ranging from applications of virtual reality to measurements of statistical association. No use of walkshed methods was reported. The authors also assessed the psychosocial outcomes of outdoor use (eg, increased social agency, anxiety, and promotion of personhood) and built environment characteristics that facilitate use and participation (eg, land use diversity, presence of landmarks, and irregular street grids).

By contrast, Sturge and colleagues [37] focused solely on qualitative studies exploring how social and built environments contribute to the well-being of persons living with dementia at home. Under a theme examining “connection to society and supportive relationships,” the authors review 4 key areas of support. These include contact with friends and family, social networks afforded by formal events and professional services, connections available across a host of neighborhood settings (eg, pubs and cafés), and the mixed reactions persons living with dementia can experience when disclosing their diagnosis. A second theme titled “interaction with natural environments and public space” examines supports (eg, parks and sounds of children playing) and barriers (eg, complex street environments and noise from traffic).

Both Peters and colleagues [2] and Akinci and colleagues [21] review (respectively) aspects of walkshed methodology in the context of older adults or older adults and the general public. Neither focused specifically on persons living with dementia. Peters and colleagues [2] distinguish between subjective and objective measures and discuss the use of accelerometers, GIS, and field-based audit approaches. They document key aspects related to the use of walkshed methods with older adults. Elements include operational definitions of a neighborhood, walking times or distances used to define a walkshed, and neighborhood attributes associated with walking and other physical activity. Akinci and colleagues [21] similarly report on GIS-based methods for spatial analysis. They report on walkshed buffer types and sizes and 167 different walkability variables across 24 studies of older adults.

The identified 6 studies are each related to the aim of this proposed scoping review. None directly cover the realm we seek to document. In 4 cases, the studies do not review objective walkshed methods. The remaining 2 cases do not focus on persons living with dementia.

## Methods

### Study Eligibility

The primary objective of this study is to report on research relevant to the use of walkshed methodology. We are specifically interested in walkshed analysis which involves the monitoring and evaluation of barriers and facilitators to walking in a neighborhood setting. Eligible studies will include those that reveal details about how to define a walkshed in a manner that is appropriate to the walking experience of persons living with dementia (eg, walking distance used to define a walkshed). We anticipate that this group of studies may largely involve quantitative, GIS-based case studies. Studies that document criteria and indicators that can be used to identify and track barriers to and facilitators of walking will also be included. We anticipate that the methodologies of these studies will be more diverse, including qualitative, quantitative, mixed methods and review articles.

We will include publications from any date in our initial pool. This may influence the variability of results, such as key definitions. A start date is not included because we anticipate that there will be a limited number of available studies related to walkshed analysis for our focus population. An open-ended start date may also allow us to identify when walkshed analysis emerged in various literatures.

We will not limit eligibility by geographic scope and will include studies from any country or region. Studies from a diverse range of geographic settings will also be eligible. This will include various community environments (eg, urban, suburban, exurban, and rural), but we anticipate that urban and suburban settings will predominate. Due to the composition of the team, eligibility will be limited to studies available in English. Our primary goal is the transfer of knowledge about rigorous methodological techniques within and beyond the academic sphere. As such, only peer-reviewed journal articles will be eligible. For additional details on study inclusion or exclusion, see Textbox 1.

**Summary of the inclusion and exclusion process and the criteria (framed as prompts) used to exclude studies.**

**Review level**

- Level 1: title, abstract, and keyword review
  Does the study include a focus on geographic areas within a community setting?
  Does the study include a focus on outdoor spaces?
  Does the study include a focus on people’s use of the community environment by walking or other forms of non-motorized mobility?
- Level 2: full text article review
  Did the study collect and analyze primary or secondary data following a structured methodological approach?
  Does the study identify measurable criteria and indicators related to walkability or report on the use of walkshed methods?
  Does the study specifically focus on environmental use by persons living with dementia or older adults?

### Population and Setting

This review will be guided by Arskey and O’Malley’s [39] 6-step scoping review process. It will include studies that involve participants recognized to be living with dementia or mild cognitive impairment and who reside in a community setting. Studies that focus on persons living in congregate care-based facilities such as assisted living homes and long-term care homes will be excluded. We expect to find few published studies that explicitly focus on this population in the context of operational aspects of walkshed methodology. As age is the primary risk factor associated with dementia, we will also include studies that focus more broadly on community-dwelling older adults aged 65 years and older [6]. We will track differences in existing evidence between these population groups.

### Search Strategy

Our search strategy was developed by a project manager with experience conducting scoping reviews. It involved consultation with a research librarian and the broader research team. The latter consultation involved a workshop that iteratively identified, tested, and respecified search domains and terms. Our search strategy includes a combination of subject headings and title or abstract-focused keyword searching (Textbox 2). These strategies target the intersection of an activity or policy domain (walking), an environmental setting domain (outdoor neighborhood setting), and a population focus domain (persons living with dementia and older adults). We will apply search strings to 6 electronic databases known to publish high-quality research around our focus domains (PubMed, Medline, CINAHL, APA PsycINFO, Business Source, and Web of Science). Endnote will be used to manage citations, and DistillerSR (DistilerSR Inc) and Excel (Microsoft Corporation) will be used to manage the inclusion, data extraction, and charting stages of this review.

**Domain areas and search terms to be used in search strings for database searches.**

**Domain areas and search terms**

- Activity or policy - walking, walkshed, walkability, walk, wayfinding, way finding, indicator, criteria, dimension, requirement, experience, audit, measure
- Environmental setting - footpath, greenspace, green space, population density, rural population, neighbourhood characteristics, city planning, communit*, neighbo*rhood*, built environment, urban design*, urban planning, town planning, city planning, building densit*, social densit*, population densit*
- Population focus - dementia, alzheimer*, aged

### Article Selection Process

After removing duplicate sources from our initial study pool using DistillerSR, we will use DistillerSR to complete screening at 2 levels. At level 1, we will assess the title, abstract, and keywords of each potential source. This assessment will include 2 independent reviewers using the level 1 inclusion criteria in Textbox 1. Studies will be excluded if both reviewers definitively identify relevant content and answer no to any of the criteria prompts. Studies will be moved to level 2 screening if a prompt cannot be answered definitively. To promote consistency at level 2 article screening, 2 reviewers will assess the full text of all remaining sources. Studies will only be included if reviewers can definitively answer yes to all inclusion prompts. We will address discrepancies at each level at a team meeting that involves a reassessment of the source and a consensus decision made by the team.

### Data Charting and Representation

Data charting and representation will follow 2 interrelated steps outlined by Arksey and O’Malley [39]. Common practices in scoping review methodology and existing knowledge syntheses documented above informed the creation of the data charting schema listed below. Using this schema, we will develop a data matrix in Excel. This matrix will organize data and allow for the analysis of key items of information. Following guidance from Levac and colleagues [40], we will review and iteratively update the initial schema shown in Table 2 as the final study pool is examined. A total of 2 reviewers will extract data for a subset of papers (n=5). They will compare and update the schema as they reflect on processes and outcomes. Final data extraction will be completed by a single reviewer.

**Table 2:** Initial data charting schema for creation of data charting matrix.

Study details	Walkshed methods	Criteria and indicators
Title	Definition of walkability	Measurement domains reported
Lead author	GIS^a^ operationalization of walkshed	Measurement criteria reported
Year of publication	Distance or time parameter	Criteria used with persons living with dementia
Journal name	Data sources and types	Criteria used with older adults
Journal discipline (if applicable)	GIS routines (if reported)	Measurement indicators reported
Country of lead author’s institution	Population focus	GIS based indicators
Study method	N/A^b^	Data sources for indicator calculation
N/A	N/A	Method for indicator measurement or representation

^a^GIS: Geographic Information System.

^b^N/A: not applicable.

Beyond tracking the breadth (eg, diversity of methods) and location (eg, countries of origin) of literature, descriptive numerical summaries will examine 2 key topics. First, we will document the tools, data, and parameters used to define a walkshed. The review will make a contribution to the existing literature by documenting implementation approaches specific to the context of persons living with dementia. We will also compare these approaches to those used in studies of an older adult population. Second, we will chart criteria and indicators used to measure aspects of dementia-friendly neighborhood and community environments. By documenting indicators that scholars have operationalized using GIS-based analyses, we will make a key contribution to the transfer of the methodology.

The final scoping review will use descriptive results (eg, diversity of methods) represented using a combination of summary tables and figures (eg, Sankey diagrams). Limited textual information will support these visual elements. We will represent comparative results related to criteria and indicators as a larger data matrix. This matrix will visualize how researchers have operationalized indicators in GIS for the 2 populations of interest. A longer textual description will contextualize these results. Finally, using thematic analysis, we will convey synthesized themes that capture nuance lacking in the descriptive and comparative results [41-43]. We expect to highlight considerations for the use of walkshed methodology not yet documented in recent studies focused on older adults [2,21]. We also expect to identify where criteria used to assess walkability for persons living with dementia and older adults converge and diverge. The risk of bias will not be assessed. This is consistent with the broad nature of our review question and the norms identified in the development of the PRISMA-ScR (Preferred Reporting Items for Systematic Reviews and Meta-Analyses Extension for Scoping Reviews) [44,45].

## Results

The results of the study and the submission of a manuscript for peer review are expected in June 2024.

## Discussion

### Overview

Scholars from the fields of planning, public health, urban design, gerontology, and architecture have produced a wealth of evidence and guidance related to walkability. Branching out from the “general population,” studies increasingly focus on targeted population groups. These foci better recognize the social, cultural, and demographic barriers and enablers to walking that shape one’s experience of the neighborhood. The proposed scoping review will synthesize the growing evidence base with specific reference to persons living with dementia. By including relevant studies focused on an older adult population, the review will also identify where current best practice for monitoring and evaluation diverges and converges for these populations. Expected findings include a detailed breakdown of current parameters and routines used to conduct walkshed analysis. Findings will also convey criteria that can be operationalized in GIS as indicators to assess barriers and facilitators to walking in a neighborhood setting.

Studies already identified here have documented monitoring and evaluation methods relevant to walkability for persons living with dementia. Methods include interviews, community survey techniques, and field audits of the neighborhood environment. Our planned focus on GIS-based walkshed analysis will further document a highly scalable monitoring and evaluation tool and technique.

### Limitations

The final scoping review will be subject to limitations, despite following accepted methodological practice [39,40]. First, as a scoping review, there will not be a quality assessment of studies, which presents a risk of bias. Second, only English studies will be included, which will overemphasize evidence and practice from western countries. Third, we expect that the use of walkshed analysis for persons living with dementia will be an offshoot of techniques and literature focused on older adults. There may therefore be limited literature specific to persons living with dementia. To mitigate the risk of making assumptions about the transfer of methodological guidance from one population to another, we will explicitly track and compare findings across groups.

### Conclusions

As far as we are aware, the proposed scoping review will be the first to provide comprehensive methodological or technical guidance for conducting walkshed analysis specific to persons living with dementia. There are 3 target audiences for this scoping review. These include applied academic researchers in the field of public health, applied academic researchers in the fields of urban planning and design, and evidence-based practitioners across these fields. Scholars identify neighborhood environments as an upstream source of barriers and enablers that shape walking behavior and associated health and well-being cobenefits [12,17,25]. Understanding the individual and population health impacts of neighborhood environments requires the expertise of health researchers and practitioners. Understanding how neighborhood environments came to be and how to reshape them through land-use and built-form interventions requires the expertise of planners and designers. By clearly documenting methods used in walkshed analysis, our goal is to spur increased collaboration across these disciplines to enable an evidence-informed approach to improving neighborhood environments for persons living with dementia.

## Abbreviations

GIS: Geographic Information System
PRISMA-P: Preferred Reporting Items for Systematic Review and Meta-Analysis Protocols
PRISMA-ScR: Preferred Reporting Items for Systematic Reviews and Meta-Analyses Extension for Scoping Reviews

## Data Availability

The data generated and analyzed during this study will include content extracted from published, peer-reviewed journal articles. Full details about parameters, data sets, and Geographic Information System routines used in walkshed analysis, as well as a full list of associated indicators, will be reported in the scoping review publication. Additional data generated and analyzed during the study will be available from the corresponding author upon reasonable request.
